# Active learning of medical students in Taiwan: a realist evaluation

**DOI:** 10.1186/s12909-020-02392-y

**Published:** 2020-12-03

**Authors:** Chien-Da Huang, Hsu-Min Tseng, Chang-Chyi Jenq, Liang-Shiou Ou

**Affiliations:** 1 Chang Gung Medical Education Research Centre, Chang Gung Memorial Hospital, Chang Gung University, College of Medicine, Taipei, Taiwan; 2 Department of Medical Education, Chang Gung Memorial Hospital, Chang Gung University, College of Medicine, Taipei, Taiwan; 3 Department of Thoracic Medicine, Chang Gung Memorial Hospital, Chang Gung University, College of Medicine, 199 Tun Hua N. Rd, Taipei, Taiwan; 4 Department of Health Care Management, Chang Gung University, College of Medicine, Taipei, Taiwan; 5 Department of Nephrology, Chang Gung Memorial Hospital, Chang Gung University, College of Medicine, Taipei, Taiwan; 6 Department of Pediatrics, Chang Gung Memorial Hospital, Chang Gung University, College of Medicine, Taipei, Taiwan

**Keywords:** Realist evaluation, Culture, Active learning, Hierarchy, Medical students

## Abstract

**Background:**

Active learning is defined as any instructional method that engages students in the learning process. Cultural differences in learning patterns can play an important role in engagement with active learning. We aimed to examine process models of active learning to understand what works, for whom and why.

**Methods:**

Forty-eight sixth- and seventh-year medical students with experience of active learning methods were purposively selected to participate in ten group interviews. Interactions around active learning were analysed using a realist evaluation framework to unpack the ‘context-mechanism-outcome’ (CMO) configurations.

**Results:**

Three core CMO configurations, including cultural, training and individual domains, were identified. In the cultural context of a strong hierarchical culture, the mechanisms of fear prompted students to be silent (outcome) and dare not give their opinions. In the training context of teacher-student familiarity alongside teachers’ guidance, the mechanisms of learning motivation, self-regulation and enthusiasm were triggered, prompting positive learning outcomes and competencies (outcome). In the individual context of learning how to learn actively at an early stage within the medical learning environment, the mechanisms of internalisation, professional identity and stress resulted in recognising active learning and advanced preparation (outcomes).

**Conclusions:**

We identified three CMO configurations of Taiwanese medical students’ active learning. The connections among hierarchical culture, fear, teachers’ guidance, motivation, the medical environment and professional identity have been shown to affect the complex interactions of learning outcomes. Fear derived from a hierarchical culture is a concern as it is a significant and specific contextual factor, often sparking fear with negative outcomes.

## Background

Active learning comprises an interactive approach to education and training designed to engage learners as they strive to acquire and understand knowledge [1]. Active learning is grounded in constructivist learning theories [2] whereby learners interacting with their subject matter facilitates the construction and ownership of knowledge. Active learning is also related to adult learning theory [3] which is founded on the principles that effective training should be relevant, engaging, active, and learner-centred. The active learning classroom is one that de-emphasises lectures and other teacher-centred forms of instruction in favour of engaged class environments that are learner-centred. Thus, students are not empty vessels into whom faculty members pour knowledge [4, 5]. Rather, they read and learn information on their own, with their instructors acting as coaches and mentors [6].

Active learning activities, including flipped classroom, problem-based learning (PBL), teamwork, team-based learning (TBL), debates, self-reflection and case-based learning (CBL), promote students’ engagement and reflection to encourage an exploration around their own attitudes and values, and facilitate their motivation to learn and develop their skills [6–8]. Among these activities, PBL promotes thoughtful engagement, encourages analytical thinking and reasoning to foster the integration and application of knowledge and is designed around well-defined learning objectives [9]. In addition, the interactive teaching/learning methods such as TBL and CBL can impart sustainable knowledge and lead to performance change and high satisfaction among students, as compared with conventional lecture-based classes [10]. However, what we do not know is whether these claims to efficacy are equal across different culture contexts.

Culture lies at the very core of how we learn relate to others and think [11]. Understanding the deep cultural differences between Western and Eastern societies about learning and development provides us with a deeper clarity around different motivations for learning. It has been argued that students from Western cultures have a motivation for learning, which follows some key epistemological themes. By contrast, according to Li, Chinese students today have inherited a Confucianist learning tradition [12]. These different motivations for learning can be explained in terms of differences in cultural dimensions: namely collectivist vs individualist cultures. Thus, it is important to examine differences in perception and the role that various dimensions of culture play in developing preferences for active learning between Western and Eastern cultures.

Realist methodology attempts to understand how particular mechanisms (usually internal and hidden to the individual) arise within different contexts and lead to a variety of outcomes. In this study, we specifically examine active learning within a Taiwanese medical education setting through the lens of a realist evaluation by using a ‘context–mechanism–outcome (CMO)’ configuration to explore the complex interactions for the development of a transferable theoretical model of what works, for whom and why.

## Methods

### Research design

The study used a qualitative approach and data was collected using focus groups (Table 1). The study focused on the perceptions of medical students’ actual experiences of active learning. Further, it examined both the process and the factors of active learning on stakeholders’ interpretation and actions, as well as the eventual outcomes of the integrated curriculum. It aimed to understand the interactions between the contextual environment of active learning methods and the mechanisms of stakeholders’ interpretation and action around the active learning. The research was approved by the institutional review board (Chang Gung Medical Foundation Institutional Review Board, CGMF-IRB) with certification of approval (104-9723B). The research process was also regulated and supervised by CGMF-IRB.

**Table 1:** Focus group interview guide for medical students

**Opening Question**	1. Tell us what active learning means to you.
**Introductory Question**	2. What sections of the integrated curriculum do you feel most related to your understanding of active learning and why?
**Transition question**	3. Think back to your experiences in the integrated curriculum. To what extent has this improved your active learning? Which aspects? How? Why? Tell me about a specific situation to help me understand.
**Key Questions**	4. How would you compare the rigor of active learning (CBL, TBL, and PBL) with passive learning (other didactic lectures)? (Workload?)
	5. What is your role in the active learning process?
	6. In which ways did the process of active learning pose barriers to your learning? Tell me about a specific situation to help me understand.
	7. What was the most challenging part of active learning in terms of planning and preparation? Expectations? Tell me about a specific situation to help me understand.
	8. How can we improve active learning across the curriculum?
**End Question**	9. Do you have any other comments about the active learning in curriculum?

### Participants

A total of 48 participants (20 females and 28 males) were recruited, including 24 (58% male) sixth-grade students and 24 (58% male) seventh-grade students. The participants’ mean ages were 25.1 [24-31 years]. Forty-eight sixth- and seventh-year medical students with experiences of active learning methods were purposively selected to participate in ten focus group interviews. The lists of students with experiences of active learning in Chang Gung Memorial Hospital (CGMH) Linkou branch were first collected from the clerkship and internship in the department of internal medicine, then purposely selected by investigators and invited by telephone. Informed consent was obtained from all participants. Participants were informed of their right to withdraw their consent at any stage of the study without penalty. Each interview lasted approximately 1 h. All interviews were audio-recorded, and a small monetary reward (New Taiwan Dollar 500) was given to thank the participants after the interviews.

### Realist evaluation

We used a realist theoretical approach. Realist evaluation was first proposed as a way of understanding the efficacy of complex interventions by Pawson and Tilley’s in their seminal work, ‘Realistic Evaluation’ [13]. Realism [14] is a philosophical viewpoint which suggests that material and social worlds are grounded in cause-effect linkages. As such, realist evaluation is used to evaluate the impact by considering the contextual environment in terms of three key connecting elements: contexts, mechanisms and outcomes. This theory-based evaluation approach begins with a clarification of the ‘intended programme theory’ that elucidates which mechanisms (often hidden, psychological processes) are likely to operate in which context and what outcomes are subsequently likely to occur [15]. From this initial ‘intended programme theory’, an investigation into the actual mechanisms, contexts and outcomes was undertaken that enabled us to develop an ‘actual programme theory’. Through this process we understood the widest range of factors that impacted on engagement with active learning. Realist evaluation therefore is a theory-driven approach focused on understanding the mechanism of what works, for whom, in what circumstances and how programmes work (or did not work) in their contextual setting, rather than simply measuring outcomes [13, 14, 16].

### Qualitative exploration

We explored qualitatively – via group interviews – learners’ (*n* = 48) perceptions and experiences of active learning. Group interviews took advantage of group dynamics by stimulating conversations among participants [17]. The guiding principle was that the psychological processes helped people to identify, reflect on, and clarify their own views and attitudes [18]. We used groups as our methodology because of its ability to elicit group and individual responses, as well as to derive information on ‘hidden agendas’, its practical utility, and the availability of institutional expertise [19]. We also included a narrative approach to interviewing which enabled us to ground participants’ comments in real experiences [20]. To ensure a wide range of experiences, a purposive sample was used: efforts were made to recruit students with diverse backgrounds and attitudes (recruitment announced that ‘all points of view are welcome and encouraged). Following the consent, group interviews were audio-taped and conducted by staff and faculty not associated with student evaluation. Both staff and students remained anonymous throughout. Audio-tapes were later transcribed in their entirety. In addition, the research assistants took field notes during the group, including observations of group process and records of keywords, sentence fragments, and summaries of basic ideas/concepts.

### Data analysis

Data collection occurred between December 2016 and July 2017. Research assistants (SW, WHC, CYS) conducted the focus group interviews. Audio-recorded interviews were transcribed verbatim and anonymously. All interview transcripts were entered into the ATLAS software package in Chinese.

The data were analysed inductively by 4 researchers (CDH, HMT, CCJ, LSO) and 2 research assistants (WHC, CYS). We used a combination of descriptive, evaluating and causation codes for coding. The research team analysed data with a focus on CMO configurations. All researchers began by analysing the same transcripts in order to develop the initial coding framework. Following this, one researcher (WHC) repeatedly read the transcripts individually and undertook data coding. All data were discussed with the wider team of researchers, who then provided feedback and assisted in developing the coding framework. We moved back and forth between the data sources, the codes, and the realist framework to synthesize relevant aspects of the CMOs [21]. In connecting strategy, we looked for relationships connected statements and events into a coherent whole [22]. After completing the initial coding of all transcripts for CMOs in Chinese, the excerpts were translated to English. A wider team for English data (including LVM) then identified and clarified the CMO configurations. We summarized the data relevant to each CMO theory in line with the realist framework [13]. This analytic process involved all authors in several research meetings in CGMERC, examining each theory for validity to reach a consensus.

## Results

### Context 1: cultural domain: hierarchical culture (Table 2)

In the context of hierarchical culture, the mechanisms of fear/boredom emerged, prompting students to be silent and dare not to give opinions. This is a significant and specific context in Taiwan sparking fear with negative outcomes.

i Hierarchical culture (top-down criticism) (C) → fear/boredom (M) → dare not give opinions (O)
  F-Y7 (7C 1:692–694): “Sometimes when I [a student] am taking some teachers’ classes, I always feel a sense of distance (C). Maybe I feel more stressed. Maybe nobody dares to answer questions (O) when the teacher asks because they are afraid of giving wrong answers...(M)”.
  M-Y7 (7B 1:722–726): “... Some teachers actually ... well, you could feel the sense of distance when he (teacher) is teaching the class (C). He just keeps talking and talking ... talking about what he knows, or talking about what he is professional about. But we (students) feel like we are dying while we are listening (O). Maybe it’s because what he talks is too deep or too much, or something else. Or maybe his speaking is quite monotonous... (M)”.

**Table 2:** Context 1: Cultural domain: hierarchical culture

Context		Mechanism		Outcomes
Hierarchical culture (top-down criticism)		Fear Boredom		Dare not give opinions

### Context 2: training domain (Table 3): interaction between teachers and students, teacher-student familiarity and teachers’ guidance

In the contexts of interaction between teachers and students, teacher-student familiarity and teachers’ guidance, learning motivation, self-regulation and enthusiasm were triggered, prompting positive learning outcomes and competencies.

i Good interaction between teachers and students (C) → Motivation is triggered (M) → Students give their opinions (O)
  F-Y7 (7C 1:694–696): “...About some teachers’ teaching methods ... well, he [teacher] would discuss things with you [student] in a friendly way, so that you would speak more (O). He will not confront you directly and say you are wrong (C). He would tell you how to do in a better way, which helps us [students] have more courage to think and to answer (M).”
ii Teachers’/students’ familiarity for active learning (C) → self-regulation and enthusiasm (M) → improved learning outcomes (O)
  M-Y6 (6D 2:674–676): “Teacher’s enthusiasm for delivering knowledge also deeply influence his students in terms of the tone or attitude (C). These affect students’ desire for pursuing knowledge (M) and affect their learning outcome (O).”
iii Teacher’s guidance (C) → motivation and enthusiasm (M) → Positive learning competencies (O)
  M-Y7 (7A 1:167–176): “...Good teachers would teach us how to solve problems by guiding rather than by directly giving a standard answer which is already in his mind ... some teachers are still able to guide us step by step to the main issues even if we are rambling on and on and on [about our answers] (C). There is a big difference in the motives when we are talking about ‘directly giving the standard answer’ and ‘reaching the standard answer through discussion’ since the processes are very different (M).”

**Table 3:** Context 2: Training domain: interaction between teachers and students, teacher-student familiarity and teachers’ guidance

Context		Mechanism		Outcomes
Good interaction between teachers and students		Motivation		Students give their opinions
Teachers’/students’ familiarity for active learning		Self-regulation Enthusiasm		Improved learning outcomes
Teachers’ guidance		Enthusiasm Motivation		Positive learning competencies

### Context 3: individual domain (Table 4)

In the context of learning “how to learn actively” early on, in the medical learning environment, the mechanisms of internalization, physician identity and stress resulted in recognizing active learning and advanced preparation.

i Learning “how to learn actively” early on (C) → internalisation (M) → recognising active learning (O)
  M-Y7 (A1 5:1456–1492): “You just pick up one topic from the general chemistry to do CBL, to train your thinking process (O). … After that, people like us would just be able to understand systems [thinking process] ... would just be able to get connected to the system ... (M). It [the thinking process] didn’t exist in the very beginning (C).
  M-Y7 (7A 1:105–107): “… Four years ago, passive learning was still dominant in our education. We were required to keep memorising things and take exams (C). And then suddenly we were required to shift to active learning, which actually is not easy (M).”
ii Medical learning environment: External effects (peers, teachers and clinical duty) (C) → professional identity and stress (M) → preparation in advance (O)
  F-Y6 (6B 2:64–67): “But I think, if you think these things are important for taking care of patients, for example ... like how to give nutrients, or give some fluids ... (C) because I think, since we are going to be interns (M), we might need to understand something fundamental or basic ... (O).
  F-Y6 (6E 3:63–65): “Sometimes somebody would tactically use the reserve psychology on you, saying ‘How can you be so useless, of not even knowing this? (C)’ And then you would think, ‘How can I lose this game? (M)’ Then you would go and read up on it, until you really understand it (O).”

**Table 4:** Context 3: Individual domain

Context		Mechanism		Outcomes
Learning “how to learn actively” from early on		Internalisation		Recognising active learning
Medical environment: External effects (peers, teachers, and clinical duty)		Professional identity Stress		Preparation in advance

## Discussion

In this study, we identified three CMO domains of medical students’ experiences of active learning. For the first domain: the context of hierarchical culture, the mechanisms of fear/boredom emerged, prompting students to be silent and dare not give opinions. For the second training domain, in the contexts of interaction between teachers and students, teacher-student familiarity and teachers’ guidance, learning motivation, self-regulation and enthusiasm were triggered, prompting positive learning outcomes and competencies. For the third individual domain, in the context of learning “how to learn actively” from early on, and medical learning environment, the mechanisms of internalisation, professional identity and stress, resulted in recognising active learning and advanced preparation. These CMOs were synthesized into a process model of active learning.

### Cultural domain

Cultural membership, issues of authority and respect, and language proficiency were identified as having a direct influence on the clinical education process [23]. Active learning, such as student-centred and problem-based methods rooted in Western culture, may not be of a truly international nature. Its compatibility with non-Western cultures has been challenged [23, 24]. Looking at the culture domain, we can see that our society will experience more difficultly with implementing active learning. East Asian education is often referred to as Confucian-heritage education, where virtue is achieved primarily by learning from teachers and imitating their attitudes [25, 26]. Confucian culture has undoubtedly been a significant influence on all aspects of the society and unmatched by any other school of thought [27]. It is one of the most frequently cited social factors in healthcare research in East Asian countries [28] and has also significantly influenced the learning styles in medical education in these countries, which need to be questioned and understood within the complex of local cultural influences [29, 30]. Students therefore are more likely to limit their individual development by depending purely on the teacher’s teaching, and their ideas, which inhibits students from doing their own critical thinking. Moreover, students are also influenced by the oriental concept of “respecting teachers” while learning from them.

In this study, we use qualitative realist evaluation. The context of a hierarchical culture is consistent with previous research that reports similar findings, including hierarchy interfered with non-Western students’ application of PBL [31]. Uncertainty, tradition, hierarchy and achievement have often been identified as more prominent in non-Western than in Western cultures [32, 33]. We find that fear and boredom are the mechanism derived from a hierarchical culture. This is a concern as it is a significant and specific context for Taiwan, sparking fear with negative learning outcomes. This suggests a certain incongruity between active learning methods and non-Western cultures. Thus, it complicates the straightforward transfer of active learning to such cultural contexts and the globalization of active learning does not postulate uniform processes and outcomes. Culturally sensitive alternatives might be considered [31].

### Training domain

Sutkin found that excellent clinical teaching is characterized by inspiring, supporting, actively involving, and communicating with students [34]. The research has identified that medical students use dual processing to rate the effectiveness of classroom teachers; an interaction between the conscious appraisal of teaching attributes and the subconscious rating of variables that portray stereotypes [35]. With regard to the context of interaction between teachers and students that is under conscious appraisal and subconscious rating, we identified the mechanism of motivation, resulting in students giving their opinions.

In many East Asian cultures, schooling primary to secondary education is heavily influenced by a passive learning culture [36, 37], where reproducing teachers’ statement is strongly emphasized and where textbooks recommended by teachers serve as the main sources of information. Thus, there is less opportunity for active learning compared with Western learners. However, different learning contexts do not keep East Asian learners from being self-regulated. Awareness of their unique identity leads them to view learning tasks as high-stakes, and to initiate learning strategies that involve self-regulation [36]. In this study, under the context of teacher-student’s familiarity of active learning without traditional and teacher-centred education, students’ self-regulation is triggered, resulting in positive learning outcomes.

The development of active learning relies on teacher’s guidance and encouragement. Facilitator’s guidance was a crucial aspect of this process, particularly in situations when students were new to the PBL process [38]. In a Japanese context of medical education, medical students in Japan have difficulty extracting problems in PBL scenarios without instructions from teachers [39]. In fact, exposing Year 1 students to the independent learning environment of PBL without providing them with adequate guidance may, rather than promoting the development of self-directed learning skills, cause them to become severely dependent on tutors, predetermined learning objectives and on rote learning in order to ‘survive’ [40, 41].

### Individual domain

In the context of learning “how to learn actively” from early on, the mechanism of internalisation results in recognizing active learning. The lack of readiness of active learning strategies that require self-regulation is problematic in East Asian medical students [36]. They expect their teachers to instruct, and themselves to be instructed or “spoon-fed” [25]. In a Japanese study, medical students consistently rely on teachers’ explanatory lectures and have low motivation to study after a pilot progress test [42]. Student maturity has also been identified as an important factor for active participation [43]. Thus, the learning context of “how to learn actively” from early on promotes internalization of active learning. This may cause them to view learning tasks as high-stakes, and lead to the recognition of active learning strategies.

In the context of the medical learning environment, the mechanisms of physician identity and stress result in studying ahead of time, particularly in the context of clinical setting where they may experience questions testing their knowledge from seniors. External pressure comes from peers, teachers and clinical responsibilities. They learn to think, act and feel like doctors by gradually taking up meaningful activities in the clinical context which helps them to prepare for lifelong learning [44]. During this process, they will become full members of a clinical community of practice and collaborate in daily activities [45, 46]. Learners who are new to a clinical setting with external pressure from peers, teachers and clinical responsibilities are in an active struggle to manage themselves as they are in the process of constructing their professional identities in the clinical training context [47, 48]. Physician identity formation as a unique medical professional in the clinical setting leads to the perception that the medical students as physicians have to allow patients to ask them about any wide-ranging medical problems [45]. Immersion in a responsible individual role, which promotes physician identity formation in the clinical setting, causes medical students to view learning tasks as high-stakes, and to initiate learning strategies in an active learning manner [36].

#### Limitations

There are several limitations in this study. The research was conducted in a single medical institution in Taiwan by analyzing as a homogenous group, therefore all the relevant CMO configurations for Asian culture and medical schools may not have been identified. However, our CMO configurations identify important patterns that are shared with similar institutions, cultures and contexts and offer wider relevance. We only collected qualitative data, so there was a limited ability to make statements about the ‘academic’ outcome/merit of active learning. Our participants had variable exposures to all forms of active learning methods, such as PBL, CBL, and TBL, and therefore we couldn’t assume that our findings applied to all forms of active learning, which were not exhaustively studied. Finally, there were many factors affecting the quality of active learning methods, especially the facilitator role and tutorial structure. Further study on teachers’ perceptions is warranted.

## Conclusion

We identified three key CMO domains of medical students’ experiences of active learning, including cultural, training, and individual domains. The connections between hierarchical culture with fear/boredom; teachers’ guidance with motivation; and the clinical environment with professional identity have been identified as affecting learning outcomes. Fear derived from a hierarchical culture is a concern in Asian context as it is a significant and specific contextual factor, often sparking fear with negative outcomes in the learning environments.

## Data Availability

The data are kept at the Chang Gung Medical Education Research Center, Chang Gung Memorial Hospital, Chang Gung University College of Medicine, Taipei, Taiwan. Any questions or requests regarding the data can be addressed to Chien-Da Huang (cdhuang@adm.cgmh.org.tw).

## Abbreviations

CMO: Context-mechanism-outcome
PBL: Problem-based learning
TBL: Team-based learning
CBL: Case-based learning
CGMF-IRB: Chang Gung Medical Foundation Institutional Review Board
CGMH: Chang Gung Memorial Hospital
C: Context
M: Mechanism
O: Outcome
